# Translation initiation and its deregulation during tumorigenesis

**DOI:** 10.1038/sj.bjc.6600222

**Published:** 2002-04-08

**Authors:** S J Watkins, C J Norbury

**Affiliations:** Cancer Research UK Molecular Oncology Laboratory, University of Oxford, Weatherall Institute of Molecular Medicine, John Radcliffe Hospital, Oxford OX3 9DS, UK

**Keywords:** translation, translation initiation factors (eIFs), growth control, internal ribosome entry sites (IRES)

## Abstract

Regulation of protein synthesis at the level of translation initiation is fundamentally important for the control of cell proliferation under normal physiological conditions. Conversely, misregulation of protein synthesis is emerging as a major contributory factor in cancer development. Most bulk protein synthesis is initiated via recognition of the mRNA 5′ cap and subsequent recognition of the initiator AUG codon by a directional scanning mechanism. However, several key regulators of tumour development are translated by a cap-independent pathway. Here we review eukaryotic translation initiation, its regulation and the ways in which this regulation can break down during tumorigenesis.

*British Journal of Cancer* (2002) **86**, 1023–1027. DOI: 10.1038/sj/bjc/6600222 www.bjcancer.com

© 2002 Cancer Research UK

## TRANSLATION RATES AND GROWTH CONTROL

Cell growth and proliferation rates depend critically upon the rate of protein synthesis. Normal cells that are able to proliferate generally do so transiently in response to appropriate extracellular cues. Withdrawal of these growth stimuli leads to cell cycle exit associated with a marked decrease in protein synthesis. In mammalian fibroblasts, inhibiting overall protein translation by 50% is sufficient to prevent the onset of DNA replication following mitogenic stimulation. Consequently there exists a critical threshold level of total protein synthesis, which must be exceeded in order to commit each cell to a round of replication. This has been interpreted as a requirement to accumulate one or more unstable protein(s) to a predetermined level. The G1 cyclins have emerged as prime candidates for this class of regulatory proteins (reviewed in Zetterberg *et al*, 1995).

**Figure 1:**
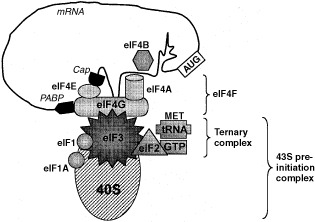
Components of the 48S translation pre-initiation complex. A polyadenylated mRNA (top) is circularised by being bound at its 5′ cap by eIF4E and at its 3′ end by poly(A)-binding protein (PABP). Both of these proteins are in turn bound to the scaffold protein eIF4G, which also provides a link to the 40S ribosomal subunit (bottom). For additional details see text.

The rate of synthesis of any given protein is determined primarily by the level of translation initiation. In mammalian cells, this is a complex process that requires collaboration between multiple eukaryotic initiation factors (eIFs: reviewed in Pestova *et al*, 2001). In brief, a ternary complex is formed between eIF2, GTP and the initiator methionyl-tRNA (met-tRNAi), while free 40S ribosomal subunits are bound to eIF3, a large, multi-subunit, initiation factor. Free 60S ribosomal units are similarly bound to the monomeric eIF6. Together, eIF3 and eIF6 prevent premature association of the 60S and 40S ribosomal subunits. The ternary complex is transferred to eIF3/40S along with eIF1 and eIF1A, to form a 43S pre-initiation complex. eIF3 can now bind to the eIF4F complex, which is associated with the mRNA, thus linking the 40S ribosome to the mRNA and generating the 48S pre-initiation complex (Figure 1

).

eIF4F itself consists of three components: eIF4G, eIF4E, and eIF4A. eIF4G binds eIF3 and acts as a scaffold for eIF4E and eIF4A. eIF4E recognises and binds the 5′ mRNA cap structure while the RNA-dependent ATPase eIF4A is thought to unwind secondary structure in the 5′ untranslated region (UTR). eIF4B is an additional factor that may stimulate the eIF4A helicase activity and promote RNA binding. The poly(A) tail of the mRNA interacts with poly(A) binding protein (PABP), which in turn has a binding site on eIF4G allowing the mRNA to circularise. The 48S pre-initiation complex now scans downstream from the mRNA 5′ end until it encounters an AUG initiation codon. This process can only occur if the 43S complex has formed in the presence of eIF1 and eIF1A.

With the Met-tRNAi positioned at the AUG codon, eIF5 interacts with the pre-initiation complex via EIF2 and eIF3. eIF2-bound GTP is hydrolysed and eIF2-GDP is released. The hydrolysis of a second GTP bound to the initiation factor eIF5B is activated by the 60S ribosomal subunit. These two successive GTP hydrolysis events, and the simultaneous release of eIF3, are essential for the joining of the 60S subunit. A functional 80S ribosome is consequently formed and peptidyl transfer can now occur.

## REGULATION OF TRANSLATION

Mammalian translational initiation control is focused on two key steps, the formation of the ternary complex and binding of the 40S-ribosome to the 5′ mRNA cap structure.

### Regulation of ternary complex formation

The protein kinases PKR, HRI and PERK can phosphorylate serine 51 of the α subunit of eIF2 and this results in an increased affinity for eIF2B, a guanine nucleotide exchange factor. Normally, eIF2B catalyses the exchange of eIF2-bound GDP with GTP so that a new interaction with met-tRNAi can take place and the ternary complex can re-form. Phosphorylated eIF2α sequesters eIF2B, preventing the formation of additional ternary complexes and inhibiting translation initiation (Sood *et al*, 2000). eIF2α has also been shown to be cleaved during apoptotic cell death, rendering eIF2 inactive and consequently disabling the ternary complex (Marissen *et al*, 2000).

### Regulation of mRNA-binding

mRNA binding to ribosomes is generally the rate limiting step in translation initiation and consequently is a major focus for regulatory pathways. Disruption of the eIF4F complex abolishes the link between the capped mRNA and the ribosomes and drastically inhibits translation. This disruption can be a result of eIF4G cleavage (e.g. by caspase 3, in response to apoptotic signals) or sequestration into insoluble bodies (e.g. Hsp27 binding after heat shock; Cuesta *et al*, 2000; Holcik *et al*, 2000). Formation of eIF4F complexes also depends upon the presence of active eIF4E which, while it is the least abundant of the translation initiation factors, is essential for binding the pre-initiation complex to the cap structure.

Availability of eIF4E is regulated through the activities of two 4E binding proteins, 4E-BP1 and 4E-BP2. These exist in a hypophosphorylated state in quiescent cells and have the capacity to sequester eIF4E by competing with eIF4G for a common 4E-binding site. This in turn prevents assembly of the eIF4F complex and inhibits translation initiation. 4E-BP1 hyperphosphorylation at multiple amino acid residues occurs in response to growth factors such as insulin, IGF-1 and angiotensin-II and involves protein kinases including FRAP, MAP kinases, PKC, ATM and casein kinase II (reviewed by Gingras *et al*, 1999). Hyperphosphorylated 4E-BP1 dissociates from eIF4E, leaving it free to participate in eIF4F formation. Conversely, in heat shocked or mitotic cells, dephosphorylation of 4E-BP1 correlates with decreased cap-dependent translation.

The activity of eIF4E may be regulated by phosphorylation (e.g. by the MAP kinase-stimulated protein kinase, Mnk1). Low levels of eIF4E phosphorylation are correlated with reduced translation rates in quiescent and mitotic cells. However, there is not a universal relationship between eIF4E phosphorylation and increased translation (Gingras *et al*, 1999).

## CAP-INDEPENDENT TRANSLATION

In addition to the cap-dependent mechanism described above, a subset of cellular mRNAs can utilise an alternative mode of translation initiation known as internal ribosomal entry. The small ribosomal subunit can bind within the mRNA at specific internal ribosome entry sites (IRES), which then direct translation initiation from a downstream AUG. Internal ribosome entry bypasses the requirement for 5′ cap binding and consequently allows translation of specific transcripts when global protein synthesis has been inhibited, for example during mitosis or in response to stress. A number of cellular mRNAs containing IRES elements encode factors that can influence proliferation (Table 1

**Table 1:**
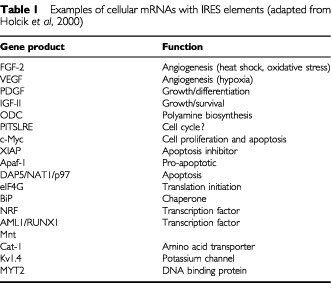
Examples of cellular mRNAs with IRES elements (adapted from Holcik *et al*, 2000)

). These include XIAP, an inhibitor of apoptosis, as well as the pro-apoptotic Apaf-1 and c-Myc. The latter can drive both cell proliferation and apoptosis and its expression is deregulated in a number of human malignancies. The angiogenic factors VEGF and FGF-2 also have transcripts containing IRES elements and promote endothelial cell growth following hypoxic stress (Holcik *et al*, 2000). These factors and their receptors are strongly implicated in cancer, with high levels linked to tumour progression, metastasis and poor prognosis.

## EUKARYOTIC TRANSLATION INITIATION FACTORS AND CANCER

A variety of lines of evidence have contributed to the emerging view that abnormal regulation of translation initiation is a widespread, and perhaps even universal, feature of tumour development.

### eIF2

Elevated expression of eIF2α has been reported in transformed cell lines. Furthermore, overexpression of eIF2α (or a mutant form which cannot be phosphorylated on serine 51) is sufficient to cause malignant transformation. Constitutively increased expression of eIF2α (together with eIF4E) is observed in non-Hodgkins lymphomas when compared with normal B-cells and correlates with disease aggression. eIF2α levels are also significantly higher in stomach, colon and rectal tumours than in normal gastrointestinal tissue (Wang *et al*, 1999; Lobo *et al*, 2000).

Down-regulation of eIF2α kinases could be comparable to the effect of up-regulating eIF2α, and reduced levels of HRI have been reported in epithelial ovarian cancers (Hwang *et al*, 2000). Conversely, PKR has been described as both a tumour suppressor and a growth promoter. PKR, however, is involved in various, alternative signaling pathways and has diverse roles (Jagus *et al*, 1999).

### eIF3

eIF3 is the largest of the eukaryotic initiation factors and comprises 11 non-identical sub-units of which five have so far been implicated in human cancer. eIF3a (p150, also known as p170) is the largest eIF3 subunit and is overexpressed in a variety of tumours when compared with normal control tissues. These include cancers of the breast, cervix, esophagus and lung (Lin *et al*, 2001; Pincheira *et al*, 2001). eIF3b (p116) has also been found significantly up-regulated in human breast carcinoma (Lin *et al*, 2001). Increased transcript levels of eIF3c (p110) were observed in all testicular seminomas examined in a recent study (Rothe *et al*, 2000). In a screen for amplified genes in breast and prostate cancer, eIF3h (p40) mRNA levels were found to be up-regulated in approximately 30% of prostate tumours and 20% of breast carcinomas (Nupponen *et al*, 1999).

Presumably, increases in expression of eIF3 subunits could lead to an increase in the amount of total eIF3, though additional biological roles for individual subunits independent of the eIF3 complex cannot be ruled out. Although eIF4E is rate limiting for cap-dependent protein synthesis, eIF3 has been reported to bind directly to the IRESs of hepatitis C virus (HCV) and classical swine fever virus (CSFV; Sizova *et al*, 1998). eIF3 may therefore have an important role in cap-independent translation of certain cellular mRNAs, which could confer a growth advantage.

Interestingly, the levels of eIF3e (p48) mRNA were recently found to be significantly reduced in approximately 40% of mammary carcinomas and 30% of non-small cell lung cancers. Murine eIF3e is encoded by Int-6, which was identified as site of mouse mammary tumour virus (MMTV) integration in mammary tumours. Consequently, disruption of this gene has been implicated in tumorigenesis (Marchetti *et al*, 2001). It has also been postulated that eIF3e/INT6 may be a negative regulator of eIF3. The subunit can interact with the interferon-inducible protein, p56 and inhibit protein synthesis *in vitro* and *in vivo* (Guo *et al*, 2000). Reduced levels of eIF3e may therefore serve to increase eIF3 activity and hence the potential for transformation.

### eIF4G

eIF4G is a large ‘scaffolding’ protein which interacts with eIF3, eIF4E, eIF4A, PABP, Mnk1, 40S ribosome and mRNA. Two related eIF4G proteins (eIF4GI and eIF4GII) exist in mammalian cells but both form eIF4F complexes capable of protein synthesis. DAP5/NAT1/p97 is a third eIF4G-like protein, strongly related to the C-terminal two thirds of eIF4G but entirely lacking the N-terminal third. Consequently it can bind eIF3 and eIF4A but not eIF4E. DAP5 is a ‘death associated protein’ and was originally isolated as a mediator of apoptotic cell death (reviewed by Gingras *et al*, 1999). However, in a recent study, over-expression of DAP5 protected neuroblastoma cells from IFNγ-induced apoptosis (Wittke *et al*, 2001). Over-expression of eIF4GI causes malignant transformation in NIH3T3 cells and increases both cap-dependent and cap-independent translation in FM3A cells (Hayashi *et al*, 2000). eIF4GI could mediate these effects by competing with 4E-BPs for binding of eIF4E and preventing inhibition of cap-dependent protein synthesis. Excess eIF4G could also bind directly to cellular IRES elements and increase translation of the growth factors and anti-apoptotic proteins discussed above. High levels of DAP5 could similarly promote cap-independent translation. In addition eIF4G mRNA itself contains an IRES, which in principle could perpetuate its own over-expression. However, increased expression of eIF4G does not appear to be common in human tumours, although amplification of the eIF4G gene has been reported in approximately 30% of squamous cell lung carcinomas (Brass *et al*, 1997).

### eIF4A

Like eIF4G, two active isoforms of eIF4A exist in mammalian cells (eIF4AI and eIF4AII) and these appear to be functionally interchangeable. Human eIF4AII is closely related to eIF4AI but their expression differs in developmental regulation and tissue specificity (Gingras *et al*, 1999). Upregulation of eIF4AI has been observed in primary hepatocellular carcinomas, where it correlated with a higher histological grading (Shuda *et al*, 2000), and in melanoma cell lines compared to normal melanocytes (Eberle *et al*, 1997).

### eIF4E

Of all the eIFs, eIF4E is the most strongly implicated in malignancy. Increased eIF4E mRNA or protein levels have been reported in carcinomas of the bladder, head and neck, liver, colon and breast (Shuda *et al*, 2000; Berkel *et al*, 2001). This is thought to reflect a primary role for eIF4E in tumorigenesis, since enforced over-expression of eIF4E *in vitro* causes malignant transformation and deregulated cell growth. Over-expression of 4E-BPI in eIF4E-transformed cells can partially reverse their tumorigenicity. Similarly, transformed rat fibroblasts expressing an antisense eIF4E mRNA are less tumorigenic when injected into mice (Gingras *et al*, 1999). Src-transformed cell lines show increased eIF4E phosphorylation (potentially enhancing the activity of the protein) as well as increased phosphorylation of 4E-BPI (Frederickson *et al*, 1991; Tuhackova *et al*, 1999).

Over-expression of eIF4E could accentuate translation of mRNAs containing long 5′ UTRs with complex secondary structure. Since eIF4E is rate limiting, various classes of mRNAs compete with each other for translation initiation, establishing an order of priority. Transcripts with unstructured 5′ UTRs are more easily bound and scanned by the pre-initiation complex and hence are preferentially translated. Transcripts with highly structured 5′ UTRs are less efficiently translated by the cap-dependent pathway and rely more on the eIF4A helicase to unwind the 5′ UTR RNA. Sequence analyses of vertebrate cDNAs have shown that those with complex 5′ UTRs include a disproportionately high number of proto-oncogene products. Many of these transcripts also contain IRES elements and are therefore more effectively translated by cap-independent protein synthesis. In contrast, mRNAs that encode housekeeping proteins rarely have highly structured 5′ UTRs (Sonenberg, 1994). eIF4E over-expression preferentially increases the synthesis of a number of oncoproteins. Cyclin D1, c-Myc, RNR2, ODC, FGF-2, and VEGF are all up-regulated in conjunction with eIF4E. Most of these factors have complex mRNA 5′ UTRs and all have been implicated in malignancy (Rosenwald *et al*, 1999; Berkel *et al*, 2001). It is likely that this is just a small number of many growth-promoting proteins whose expression is directly or indirectly regulated by eIF4E. Consequently, a healthy cell tightly regulates proteins that are necessary in specific cellular environments but which could potentially be oncogenic. With an over-abundance of eIF4E this regulation would be lost.

### eIF5A

eIF5A is unique in that it is the only cellular protein so far known to contain the amino acid hypusine. eIF5A is not required for general protein synthesis but rather to facilitate the translation of specific subsets of mRNAs. It has also been implicated in mRNA transport and stability (Wang *et al*, 2001). To date, two human isoforms of eIF5A have been identified (eIF5A1 and eIF5A2) and amplification of the eIF5A2 gene has been found in ovarian cancer (Guan *et al*, 2001). Depletion of eIF5A in *S. cerevisiae* causes cell cycle arrest at G1/S phase suggesting that it specifically directs the translation of mRNAs required for cell growth (Wang *et al*, 2001). It is conceivable that over-expression of equivalent proteins, caused by excess eIF5A in human cells, could enhance cell growth and contribute to tumorigenesis.

## CONCLUSIONS

Early kinetic analyses suggested that normal quiescent cells requires an increase in global protein synthesis to enter the cell cycle. According to this view, an unscheduled increase in general translation would accelerate cell growth and could lead to unscheduled cell cycle entry. Misregulation of translation factors could therefore contribute to tumorigenesis simply via induction of superfluous protein synthesis. Alternatively, more subtle alterations in translational control of selective mRNA subsets could be fundamental to the involvement of translation factors in tumorigenesis. Given the complexity of cancer biology, a more realistic view might involve a combination of both of these mechanisms.

eIF4E over-expression is consistently found in a wide range of human tumours (Table 2

**Table 2:**
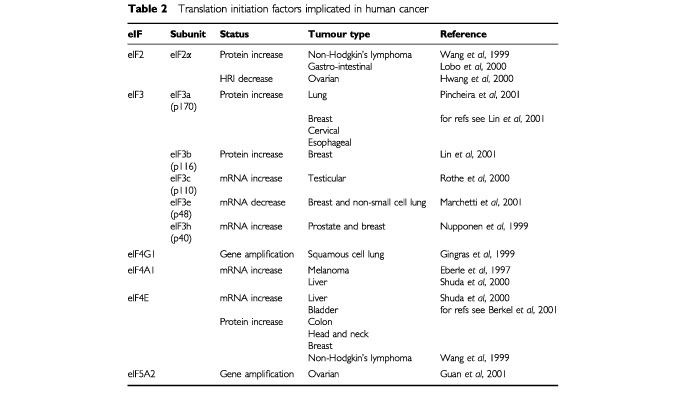
Translation initiation factors implicated in human cancer

) and is sufficient to cause malignant transformation in experimental models. Likewise, surplus eIF2α may exceed the phosphorylation capacity of eIF2α kinases and allow ternary complex formation, despite negative regulation. eIF4G and eIF3 have the potential to bind directly to mRNA, hence allowing additional translation from IRESs. Overexpression of eIF4A could increase the unwinding of mRNAs with highly structured 5′ UTRs and allow more efficient translation of these transcripts. In each case an element of translational control would be lost.

If misregulation of translation initiation is a common feature of tumorigenesis, might this generality lead to the identification of new drug targets? Certainly, a variety of agents that inhibit translation initiation have anticancer activity in experimental models. Eicosapentaenoic acid (EPA), clotrimazole and thiazolidinediones inhibit the growth of several cancer cell lines and can also reduce tumour growth *in vivo*. All act by releasing Ca^2+^ ions from intracellular stores and in this manner stimulate PKR. Hence, translation is inhibited by phosphorylation of eIF2α and cells are arrested in G_0_ (Palakurthi *et al*, 2000, 2001). Flavonoids, such as quercetin and genistein, activate all three eIF2α kinases (PKR, HRI and PERK) and can arrest the growth of leukaemia cells *in vitro* (Ito *et al*, 1999). Rapamycin and rapamycin analogues inhibit proliferation in a variety of tumour cell lines (Hidalgo and Rowinsky, 2000). Rapamycin acts by blocking the protein kinase activity of FRAP/mTOR, which is ordinarily responsible for phosphorylating 4E-BP1. With 4E-BP1 phosphorylation inhibited, eIF4E is not released and cap-dependent translation is repressed. Although these compounds have promising anticancer activities, from a theoretical standpoint it is not clear why they might have general tumour cell selectivity; inhibition of translation would be predicted to result in broadly non-specific toxicity. Nonetheless, future developments in this area promise not only to illuminate basic cancer biology but also to offer exciting new therapeutic tools.
